# Which Is Better: Holdout or Full-Sample Classifier Design?

**DOI:** 10.1155/2008/297945

**Published:** 2007-12-12

**Authors:** Marcel Brun, Qian Xu, Edward R Dougherty

**Affiliations:** 1 Computational Biology Division, Translational Genomics Research Institute, Phoenix, AZ 85004, USA; 2 Department of Electrical and Computer Engineering, Texas A&M University, College Station, TX 77843, USA

## Abstract

Is it better to design a classifier and estimate its error on the full sample or to design a classifier on a training subset and estimate its error on the holdout test subset? Full-sample design provides the better classifier; nevertheless, one might choose holdout with the hope of better error estimation. A conservative criterion to decide the best course is to aim at a classifier whose error is less than a given bound. Then the choice between full-sample and holdout designs depends on which possesses the smaller expected bound. Using this criterion, we examine the choice between holdout and several full-sample error estimators using covariance models and a patient-data model. Full-sample design consistently outperforms holdout design. The relation between the two designs is revealed via a decomposition of the expected bound into the sum of the expected true error and the expected conditional standard deviation of the true error.

## 1. Introduction

In most microarray-based classification studies, the number of data points (microarrays) is very small (under 100) and one has no choice but to use the full cohort of data for both training and testing (error estimation). One must choose among error estimators for which the full sample is used for training. In small-sample situations, these estimators usually suffer from either low bias (resubstitution) or high variance (cross-validation) [1,2]. Studies indicate that either bootstrap [3] or bolstering [4] tend to provide better estimation. But what happens when samples sizes are not so small, a situation that will become more common as technology improves? Then, rather than using full-sample design and estimation, one has the option of holding out data from the design and using the holdout data for estimating the error of the classifier designed on the data not held out.

Based upon colloquial discussions, it appears that some people prefer to hold out data except for very small samples, thereby splitting the sample into training and testing data; however, these discussions usually lack any precise statistical justification. On the other hand, when discussing holding out test data to estimate the error of a designed classifier, Devroye et al. state [5], "A serious problem concerning the practical applicability of the [hold-out] estimate introduced above is that it requires a large, independent testing sequence. In practice, however, an additional sample is rarely available. One usually wants to incorporate all available [sample points]  pairs in the decision function." When made by premier pattern-recognition researchers such as L. Devroye, L. Gyorfi, and G. Lugosi, such a statement should give pause to anyone taking a counter position. The holdout issue arises because, even though we are assured of a smaller true error using full-sample design, we desire a satisfactory estimate of the error. The salient word in the Devroye et al. quote [5] is "rarely." Reasoning in a hyperbolic extreme, if there were an infinite amount of data, it could be split into infinite training and test data sets and this would constitute one of the rare cases. But why do so? For many popular full-sample error estimators, the mean-square error between the estimated and true errors goes to  as the sample size tends to infinity. For instance, for the histogram rule with  cells, the resubstitution estimator is low biased; nevertheless, it satisfies the bound , where  and  are the estimated and true errors, respectively [5]. In the other direction, if one has only  sample points, then clearly one does not want to hold out data from training. But what is the preferred course of action in moderate cases. Since these are not rare, are we to conclude from the Devroye et al. statement that even in these one should not hold out data for error estimation?

Let us motivate the issue with an illustration of the kind of pathology that can afflict holdout error estimation. Suppose that one randomly splits the available data in the sample, , into training and test data samples, say  and , respectively. Let  and  be the classifiers trained on  and , respectively. Now suppose that  provides a faithful sampling of the feature-label distribution, at least to the extent possible given the size of the sample; however, owing to chance in the splitting process,  and  represent different parts of the feature-label distribution. Since  provides a representative sample,  should provide good classification and this will likely be reflected in its estimated error based on . On the other hand,  may or may not provide good classification, depending on how well  reflects the feature-label distribution, but in either event, its estimated error will likely indicate poor performance because the estimate will be done on data significantly different from the training data. Splitting the data has had two undesirable effects: poorer design and poorer error estimation. The latter effect is pernicious: one has the data to design a good classifier, and indeed may even do so, but gets a high test-data error and mistakenly walks away with nothing.

One might argue that, owing to the high variance associated with many full-sample error estimators, it is more conservative, and thus safer, to split the data. But even if we desire conservativeness, this argument requires refinement. The empirical test-data error estimator also has variance, which is substantial for small test-data sets. Hence, to be meaningful, the conservative holdout argument requires a specification of the proportion of data to be held out.

Stating the matter quantitatively, given a sample  of size , is it better to design a classifier and estimate its error on the full sample  or take a holdout approach by designing on a training subset  of size  and testing on a disjoint subset  of size , where  Letting  and  denote the classifiers designed using full-sample and holdout, respectively, then the expected error of  on the full feature-label distribution is less than the expected error of  on the full feature-label distribution: , where  denotes classifier error. Were we able to compute the true error of a designed classifier, there would be no issue: design on the full sample. In practice, this error must be estimated and therefore we must concern ourselves with the relation between the error estimates  and  for  and , respectively, where  is obtained by some full-sample method and  is the error rate of  on the test data. If  is approximately unbiased, meaning that , then since  is unbiased, on average the full-sample-and test-sample-based estimators agree with the true errors of the classifiers they are estimating; however, if one of the estimators has a much greater variance than the other, say, the variance of  is large in comparison to , then we have greater confidence in the estimated error of a particular training-data designed classifier than the error of the corresponding particular full-sample designed classifier. Since holding out a significant amount of data usually means that , it is common to trust the holdout estimate over the full-sample estimate. This conservative approach has a price, that being poorer performing classifiers.

To get at the key practical dilemma facing holdout design, consider a situation in which one has  data points and wishes to split the data into training and test sets. With  given, how is one to choose ? Unless this question is to be answered in an ad hoc manner, there needs to be a criterion. A very conservative way to proceed is to take a minimax approach and choose  so as to minimize the maximum variance of the estimator. While certainly rigorous, this minimax criterion leads to the decision : the training data consists of one point from each class and the resulting classifier is tested on the  points held out. No one would opt for this minimax criterion on the variance because the expected error of the designed classifier would be very large. One would have an excellent error estimate for a useless classifier.

To unravel the problem of choosing between full-sample and holdout design, we must consider what we are trying to accomplish. Assuming that we are using an approximately unbiased full-sample estimator, a simplistic view of the matter is that we use full-sample design if the main goal is a better classifier and holdout if the main goal is better error estimation. Such a methodological choice is dependent on the properties of the design-test process, not on the result of a particular design. It is certainly possible that for a given sample,  or that . These relations cannot be known from the sample at hand. One chooses the holdout error estimator because (for sufficiently large ) its expected absolute (or square) deviation from the true error is less than the expected absolute (or square) deviation of full-sample error estimator from the true error,(1)

But this relation alone does not provide a good criterion for making the choice since, in analogy with the minimax approach to holdout, the inequality can best be achieved by letting . We are in the conundrum because the criterion of the choice, either better classifier design or better error estimation, is wrong. We want good classifier design *and* good error estimation, so the choice should be based on a criterion that takes the full process, design and error estimation, into account, not just one or the other.

In proposing a criterion, we take the conservative perspective that we want a classifier whose error is not too large, below some tolerance bound. Given random sampling, at best we can have some confidence, say , that a bound is satisfied. This calls for specifying  one-sided confidence intervals for the true errors  and  based on the estimates  and , respectively. This gives rise to two conditional confidence intervals, a  conditional confidence interval  for the true error  of the full-sample designed classifier, where(2)

and a  conditional confidence interval  for the true error  of the training-sample designed classifier, where(3)

Whereas the estimates themselves contain no information regarding their imprecision, the confidence intervals do. Since we have equal confidence in both intervals,  and , the better classifier is the one possessing the smaller confidence bound. Under this criterion, the choice between full-sample and holdout design becomes a choice as to which is smaller,  or  .

To obtain a proper criterion, the estimators must take into account the dependence of the designed classifiers on the random samples, not simply a particular sample. Hence, our real interest is in comparing  and , where the expectations are taken with respect to the appropriate spaces of samples. These expectations can be expressed as(4)(5)

where  and  are the densities for the estimation values  and , respectively, and we use  in both integrals because in this context it is a dummy variable. M is used to denote a mean because  and  are the means of the bounds  and , respectively.

Given that a full-sample error estimator is close to being unbiased, the criterion is to choose full-sample design if and only if , where the decision depends on , , and the full-sample estimator (as well as the classification rule and feature-label distribution). As we will see in the examples, it does not appear that the relation is sensitive to the choice of . We emphasize that we only apply the confidence-bound criterion when the error estimator is not strongly biased. In particular, we will not apply it when using resubstitution because we wish to avoid situations in which we expect that the error estimate is low; indeed, the criterion is reasonable precisely because it incorporates variance information to discriminate between approximately unbiased estimators.

## 2. Systems and Mthods

Using simulations we will compare  and  for several data models and classification rules. The classification rules used are 3-nearest neighbor (NN), linear discriminant analysis (LDA), quadratic discriminant analysis (QDA), and Gaussian Kernel (Kernel).

The estimators considered are leave-one-out cross validation (Loo), 5-fold cross-validation with 20 replications (CV), 0.632-bootstrap (B632), bolstered resubstitution (Bolster), and semi-bolstered resubstitution (S-Bolster) [4]. For the computation of CV we use stratified cross-validation, whereby the classes are represented in each fold by the same proportion as in the original data. For the computation of the B632 estimator we use a technique called balanced bootstrap resampling [6], where each sample point is made to appear  times in the computation. For bolstering estimators, 10 Monte Carlo samples are used for each bolstering kernel.

### 2.1. Model-Based Simulation

Simulated data consists in  points of dimension, generated randomly from three different two-classes models:

Linear Model (0)

The class-conditional distributions  and  of the points  for classes  and , respectively, are Gaussian with identical covariance matrices  (the structure of  to be specified) and means  and :(6)

The Bayes classifier is linear and its decision boundary is a hyperplane.

Nonlinear Model (1)

This is similar to the previous model, but the covariance matrices differ by a scaling factor such that . Throughout the study we use . The Bayes classifier is nonlinear and its decision boundary is quadratic.

Bimodal Model (2)

The class-conditional distribution of class  is Gaussian with mean  and the class-conditional distribution of class  is a mixture of two equiprobable Gaussians,(7)

where  and  are defined by (6), with means at  and , respectively. All of the Gaussians possess identical covariance matrices, .

As in a number of other studies [7–10], we use a block structure for the covariance matrices that models a feature set partitioned so that the features in a partition are correlated and features in different partitions are uncorrelated. All features have common variance, so that the  diagonal elements have identical value . To set the correlations between features, the  features are equally divided into  groups, with each group having  features. Possible values of  are . Features from different groups are uncorrelated and features from the same group possess the same correlation . When , all the features are uncorrelated. Values of , and  are used in the simulations, varying the amount of confusion and redundancy between the variables.

An special case is considered when using feature selection, being  the number of features the classifier will use. The values used are  or . When  there is no feature selection. Otherwise, there is feature selection, and the error is estimated using the design described in [11] to avoid bias introduced by the feature selection process. In each case, the best features were obtained by applying statistical *t-test* and selecting the features with the lowest *p-value*.

Rather than considering a covariance matrix with a fixed value , for which the Bayes error will also be fixed, we can let  vary, thereby letting the Bayes error vary, thereby emulating the practical situation in which methods are applied to classification problems of varying difficulty. To do this, we assume that the Bayes error can be any value between  and  and that it obeys a Beta distribution . The expected Bayes error is . In our simulation, we use the values  and . These generate six pairs  and the corresponding expected Bayes errors : .

To simulate models with specified Bayes errors, a table of the Bayes error for each value of , covariance matrix structure, and variance  is constructed using Monte Carlo simulations, assuming no feature selection. Six sets of simulations, or *experiments*, are used to analyze the performance of the holdout approach against full-sample approaches. Each experiment is used to compare the expected bounds across different conditions: experiment  tests all the classification rules listed in Section 2; experiment  tests a combination of different models and different values for the parameter ; experiment  tests a combination of different values for both  and ; experiment  tests a combination of different models and different number of groups ; experiment  studies the influence of the partition size on the error rates; and experiment  studies the influence of feature selection. Table 1 shows the parameters used for the six experiments.

**Table 1:** List of experiments and their parameters: **** and **** are the parameters of the Beta distribution used for the Bayes error,  is the number of groups, **** is the classification algorithm, **** is the two-classes model,  is the correlation for features in the same group, **** is the number of training samples, **** is the number of features, and  is the number of features used by the classifier.

								
	1	1	2	NN	1	0.125	100	(10,10)
				LDA				
				QDA				
				Kernel				
	1	1	2	NN			100	(10,10)
			2	NN	1	0.125	100	(10,10)
	1	1		NN		0.125	100	(10,10)
	1	1	2	NN	1	0.125		(10,10)
	1	1	5	LDA	1	0.125	100	(10,10)
				(10,5)				
				(25,5)				
				(50,5)				
				(100,5)				

In all cases we use a fixed sample size . Additional results and experiments are available at http://www.ece.tamu.edu/~edward/holdout.

### 2.2. Patient Data

In addition to the covariance models, we consider a model based on a microarray classification study. The microarrays were prepared with RNA from 295 breast cancer patients [12]. Using a previously established 70-gene prognosis profile [13], a prognosis signature based on gene-expression was proposed that correlates well with patient survival data and other existing clinical measures. Of the 295 microarrays, 115 belong to the "good prognosis" class (label 1) and the remaining 180 belong to the "poor prognosis" class (label 0). Each data point is a 70-expression vector corresponding to a single microarray, with expression values being log intensity. The best 2-gene sets for linear classification (LDA) were obtained using a full search [14] and have been selected for this analysis. The data are available at the supplementary data web page cited in [14].

**Figure 1:**
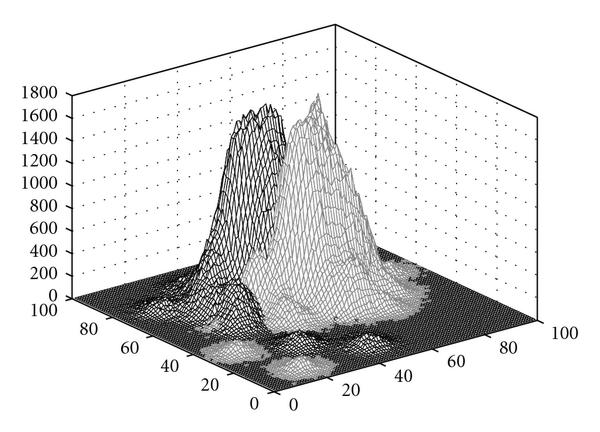
**Marginal distributions for the two classes**.

From the data, we generate a Gaussian distribution at each of the 295 points, with the variances computed for each class using the method in [4]. These are combined according to class to produce two conditional distributions (Figure 1). For each feature set, we select  training points for holdout, leaving  points for the holdout testing. To achieve good full-sample error estimation, bolstered resubstitution is done over the  sample points. We use more than 1 000 000 sample points from the distribution to accurately estimate the true error. The procedure is replicated 10 000 times.

### 2.3. Estimation

The expectations in (4) and (5) are estimated from sample data drawn from the previously defined models. A sample point consists of a feature vector  and a label , the pairs  possessing a joint distribution . A sample  of size  is split into a training set  of  independent observations and test set  of  independent observations. A classification rule  maps a dataset  into a *designed* classifier: . The true error of a designed classifier  is its error rate for the joint distribution :(8)

The true error is estimated using a large additional dataset (above 2000 samples) sampled from the distribution .

The simulation first generates the Bayes error given the Beta distribution and the value of the variance  is taken from a table of Bayes error versus variance. A set  of size  is drawn from the feature-label distribution  and split in two sets  and  for the holdout analysis. Each classification rule  (and the feature selection algorithm, when needed) is applied to both  and  to obtain the classifiers  and  (and the list of selected features when FS is applied). These classifiers are applied to  test points independently sampled from  and the average error rates are used as the true errors  and . Holdout error estimation is accomplished by applying the classifier  to the holdout sample  to obtain the holdout estimated error  as the proportion of errors  makes on . Full-sample error estimation for each method is evaluated using the whole set  to obtain the estimated error . When feature selection is used, each classifier design involves feature selection. For resampling techniques it involves an additional cost for the process, since FS is applied to each iteration.

This procedure is repeated  times ( times for experiment ) to obtain  pairs  and , which provide tight approximations to the joint distributions  and . From these we compute the  upper-confidence bounds  and , and from these the expected upper-confidence bounds  and , where the expectations are relative to the distributions of the estimated errors  and , respectively.

**Figure 2:**
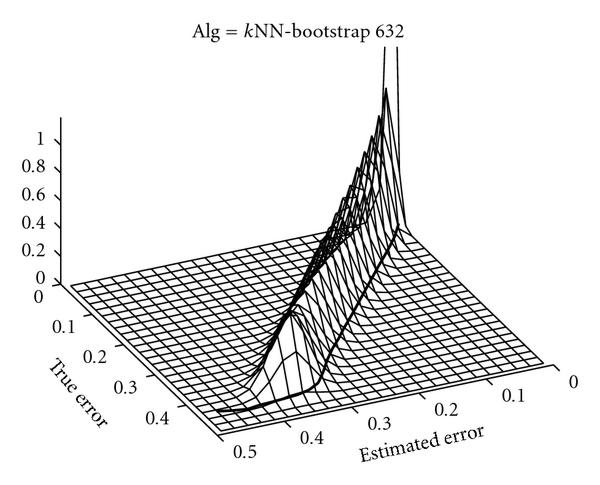
**Examples of joint distribution between true error and estimated error**. The black line shows the threshold  as function of the estimated error .

Figure 2 shows an example of the estimated joint distribution  for  of the true and full-sample estimated errors when  is based on NN and the error estimation is .632 bootstrap. The solid line in the figure represents the upper bound for the  confidence interval, defined by , , as a function of the estimated error . Equations (2) and (3) define the expected values of this upper bound when using full-sample and holdout error estimation, respectively.

## 3. Results and Discussion

### 3.1. Quantitative Results

**Figure 3:**
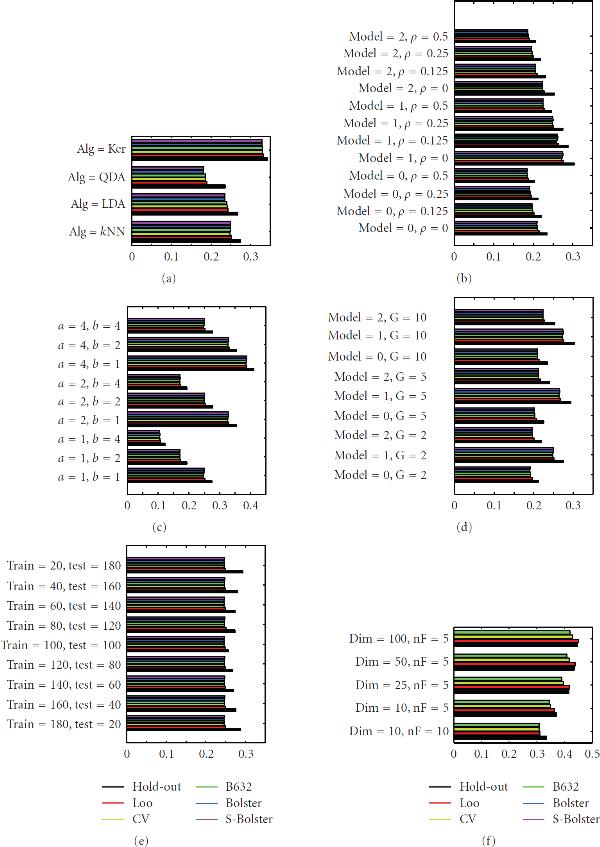
**Expected 95% bounds for true error for experiments , , , , , and  (a) to (f), resp.)**.

The model-based experimental results are displayed in Figure 3, parts (a) through (f) corresponding to experiments  through , respectively, with the bars giving the expected  confidence bounds for the true errors.

Tables available at http://www.ece.tamu.edu/~edward/holdout. provide the actual numerical values. In all cases, holdout error estimation has the highest expected  bound, meaning that holdout error estimator is outperformed by the full-sample error estimators. Among the latter, leave-one-out cross-validation generally performs the worst.

**Figure 4:**
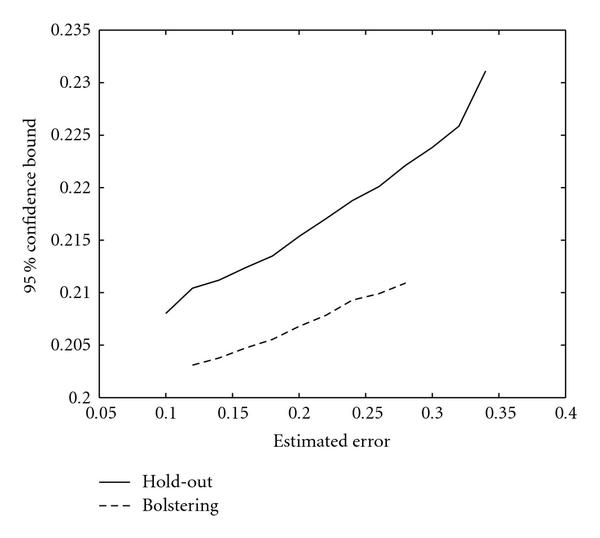
**95% bounds for true error for patient data**.

Confidence bound graphs for the patient data are shown in Figure 4. The full-training method yields lower bounds than does the holdout. The expected  bounds for the true error are  and  for holdout and bolstered resubstitution, respectively.

### 3.2. Analysis

Holdout forces one to make a choice between low variance and good performance, and this turns out to be a classical "dammed if you do, dammed if you do not" decision. This conundrum can be analytically expressed if we assume that, given the estimated error, the true error is normally distributed. Letting  and  denote the true and estimate errors, without regard to the design and testing procedures, the equation for the confidence bound becomes(9)

where  denotes the bound for the  conditional confidence interval. This expression can be written as(10)

in which form we recognize that the confidence interval is for , the true error given the estimated error. Assuming that  is normally distributed, the probability expression can be written as(11)

where  is the standard normal variable,  is the conditional expectation of  given , and  is the conditional standard deviation of  given . If  is approximately normally distributed, then the relation is approximate. If we let  denote the  upper bound for the standard normal variable, meaning , then the preceding equation implies(12)

If we now take the expectation with respect to , we obtain(13)

Finally, since , we obtain(14)

Equation (14) quantifies the dichotomy between opting for better error estimation or better actual performance.

Rather than using (4) and (5), we can express  and  via (14). To do so, let  and  denote the error and estimated error using full-sample design, and let  and  denote the error and estimated error using holdout design. Then, according to (14),(15)(16)

According to (16), a large holdout reduces  at the cost of increasing . Indeed, large  decreases  at the cost of increasing  and small  decreases  at the cost of increasing . The combined effect is seen in Figure 3(e), where for increasing ,  first decreases and then increases. This effect can also be seen for QDA in similar graphs available at http://www.ece.tamu.edu/~edward/holdout. None of this should make us lose sight of the main observation: in all cases, both for 3NN and QDA, holdout performs worse than the full-sample estimators.

**Figure 5:**
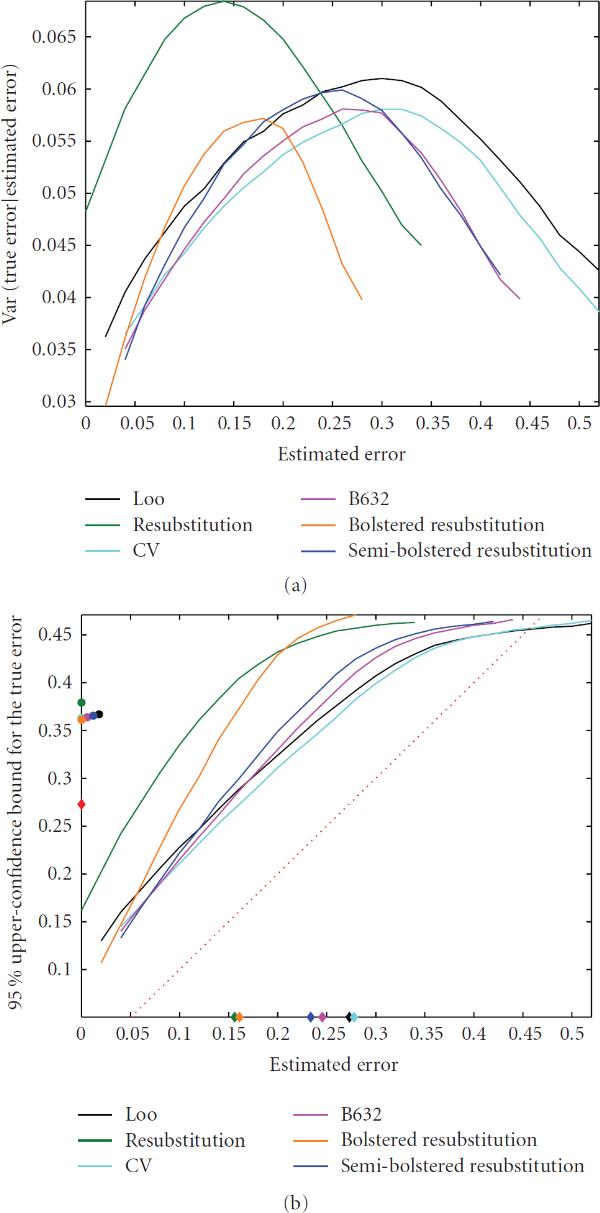
**(a) Conditional variance for the true error for 3NN**; (b) conditional 95% bounds for the true error for 3NN.

Perhaps what is most interesting about (14) is the manner in which the variance manifests itself. It is not the standard deviation of the estimate; rather, it is the expected conditional standard deviation of the true error given the estimate. To help explain the implications of this observation, we will consider resubstution estimation. Although we would not use the confidence-bound analysis for resubstitution owing to its usual low bias, we can certainly compute  for resubstitution, and we believe that doing so is enlightening. The variance of resubstitution is significantly less than that of cross-validation in the cases studied [1]; however,  is generally larger for resubstitution than for cross-validation (see table of resubstitution values available at http://www.ece.tamu.edu/~edward/holdout). Given the approximation of (14), this can only be the result of the conditional variance term because  and  are common to both error estimators; that is,  is greater for resubstitution than it is for cross-validation. This phenomenon is illustrated for 3NN in Figure 5. Figure 5(a) shows the conditional-variance curves for  for the nonlinear model, with 2 feature groups, feature correlation , and expected Bayes error , and Figure 5(b) shows the corresponding conditional confidence bounds. In Figure 5(b), the means of the estimated errors are marked on the horizonal axis, the means of the  confidence bounds are marked on the vertical axis, and the mean true error is marked on the vertical axis by a red diamond. It is clear that the resubstitution conditional variance is greater near its center of mass than are the other estimators near their centers of mass, thereby leading to a greater expected conditional standard deviation for resubstitution and thus a greater expected confidence bound for resubstitution.

The appearance of the expected conditional standard deviation of the true error in the partition of  in (14) is not counterintuitive. If we assume that the error estimator is unbiased, then . If we now assume that  is small, then  is small relative to the distributional mass of , which in turn means that  relative to the mass of , which then implies that  is small; that is, the error estimator is performing well.

### 3.3. Concluding Remarks

We propose a confidence-based criterion to decide between experimental designs, our particular interest being between full-sample and holdout classifier designs. One is free to propose other criteria, but reasonable probabilistic criteria upon which to ground a decision are certainly needed. Given the importance of the applications being considered, to leave matters in an ad hoc state of affairs is unacceptable. A critical point of the experiments is that the decision for full-sample design holds across various models and parametric settings, and the decision is generally clear cut. This consistency is important for practical application, where one does not know the feature-label models.
